# Edge-Aware Attention with Shallow-Guided Fusion for Ship Detection in Remote Sensing

**DOI:** 10.3390/s26185821

**Published:** 2026-09-14

**Authors:** Haiyang He, Liang Dong

**Affiliations:** School of Electrical Engineering, Liaoning University of Technology, Jinzhou 121001, China; 240384034@stu.lnut.edu.cn

**Keywords:** remote sensing ship detection, RT-DETR-s, edge-aware self-attention, shallow-guided deep fusion

## Abstract

Remote sensing ship detection is of great significance in marine traffic monitoring, port management, national defense security and other fields. Due to the small target scale, complex backgrounds and sparse distribution of ships in remote sensing images, existing real-time detectors still face challenges in localization accuracy and feature utilization efficiency. Taking RT-DETR-s as the baseline, this paper proposes two encoder-level improvements to boost the performance of ship detection in remote sensing imagery. On the attention mechanism side, an Edge-Aware Self-Attention (EASA) module is proposed as the principal methodological contribution. The Scharr edge map is injected into the attention matrix before softmax with a learnable intensity, providing a structural prior that enhances attention interactions between positions with strong edge responses, including ship contours, coastlines, and wake boundaries. The semantic distinction between ship-relevant and irrelevant edges is learned through the query-key projections in the attention mechanism itself, rather than through the edge bias. In terms of feature fusion, a Shallow-Guided Deep Fusion (SGDF) module is designed as an efficient integration strategy of existing techniques (CBAM attention and cross-scale fusion), with its novelty in the attention-first, downsampling-later pipeline that preserves fine-grained spatial details for small-object detection. It injects high-resolution small-object context from the backbone’s shallow layers into deep features, effectively mitigating the loss of spatial information of small ships in deep feature maps via spatial-channel attention. Finally, the existing WIoUv3 loss function is adopted and systematically compared with six other IoU variants to identify the most suitable regression loss for remote sensing ship detection. Experiments demonstrate that compared with RT-DETR-s, the proposed method achieves 2.8% higher mAP50–95 and 3.2% higher APsmall (APs) while maintaining real-time inference speed (37.6 FPS).

## 1. Introduction

As a core task of intelligent interpretation for remote sensing images, remote sensing ship detection has wide applications in marine traffic monitoring, port scheduling management, fishery resource investigation, national defense early warning and other scenarios [1,2]. With the rapid development of high-resolution remote sensing satellites and aerial remote sensing platforms, the volume of acquired remote sensing image data has exploded, raising urgent demands for automated, high-precision ship detection algorithms. Nevertheless, ship detection from remote sensing imagery encounters multiple difficulties: first, ships generally appear as small-scale targets whose spatial information is easily lost after multiple downsampling operations in deep feature maps; second, remote sensing scenes contain cluttered backgrounds including ocean clutter, port buildings and coastlines that interfere with target localization; third, practical deployment imposes strict constraints on inference speed, requiring a balance between precision and efficiency [3,4].

In recent years, deep learning-based detection methods have achieved remarkable progress in remote sensing applications. Two-stage detectors such as Faster R-CNN [5] generate candidate boxes via region proposal networks with high precision but suffer from slow inference speed, failing real-time requirements. Single-stage detectors, including the YOLO series [6] and SSD [7], directly regress bounding boxes to accelerate inference. However, CNN-based detectors are limited by local receptive fields and lack the capacity to model global context. DETR [8] first introduces the Transformer into object detection, establishing an end-to-end paradigm via self-attention for global feature interaction. On this basis, Co-DETR [9] improves encoder feature learning through collaborative mixed assignment training, while DDQ [10] boosts efficiency via dense query selection while retaining end-to-end properties. The RT-DETR [11] series is optimized for real-time detection scenarios, and RF-DETR [12] further pushes real-time detection accuracy via neural architecture search. Even so, RT-DETR still leaves room for optimization of encoder feature utilization, especially for small ship detection in remote sensing imagery.

This paper addresses two critical issues in remote sensing ship detection: insufficient localization accuracy for small ships and severe spatial information loss in deep features. In remote sensing images, small ships occupy only a handful of pixels after downsampling, leading to heavy attenuation of edge and fine details. Conventional self-attention computes weights solely based on semantic similarity and cannot distinguish ship edges from flat ocean backgrounds, restricting small-object localization accuracy. Additionally, high-resolution spatial details preserved in shallow backbone layers are not effectively fused into deep semantic features, exacerbating context loss for small ships. Aiming at the above drawbacks, we propose two improvement strategies at the attention and feature fusion levels with RT-DETR-s as the baseline, and validate the effectiveness of the proposed framework on remote sensing ship datasets.

The main contributions of this work are summarized as follows:

1. An EASA module is proposed as the principal methodological contribution, which embeds Scharr edge maps into the self-attention computation prior to softmax. Unlike post-hoc gating modulation, this method internalizes a structural edge prior into core attention calculation. The edge bias enhances attention to boundary regions in general, while the learned query-key projections provide the semantic selectivity to prioritize ship-relevant edges over other scene boundaries, collectively delivering improved localization precision.
2. A SGDF module is developed as an efficient integration strategy of existing techniques (CBAM attention and cross-scale fusion). It extracts small-object context from high-resolution shallow layers through an “attention-first, downsampling-later” pipeline before cross-scale fusion, elevating detection rates for small-scale ships.
3. The existing WIoUv3 loss function is adopted and systematically compared with six other IoU variants to identify the most suitable regression loss for remote sensing ship detection.
4. Sufficient ablation and comparison experiments are conducted on remote sensing ship datasets. Ablation studies verify the independent performance gain brought by EASA and SGDF. When combined, the two modules significantly boost small-object detection accuracy while retaining real-time inference speed to the baseline RT-DETR-s.

The source code is available at https://github.com/hhy-98/improve-RT-DETR, accessed on 9 September 2026.

## 2. Related Work

### 2.1. Remote Sensing Object Detection and Real-Time Detectors

Early remote sensing ship detection relies on handcrafted features such as HOG [13] and LBP [14] combined with SVM [15] classifiers, which suffer from limited generalization capacity over complex remote sensing backgrounds. Deep CNNs have gradually become the mainstream solution. Two-stage architectures like Faster R-CNN exploit multi-scale feature extraction for high accuracy yet incur heavy computation overhead. Single-stage detectors SSD and YOLOv3 accelerate inference via dense sampling but underperform on tiny targets. D-FINE [16] redefines bounding box regression via fine-grained distribution optimization, and DEIM [17] accelerates DETR convergence through dense one-to-one matching. Nevertheless, most of these networks are designed for generic natural images, and their performance on remote sensing ship detection remains to be verified.

The success of Transformer [18] in NLP has inspired its adoption in computer vision. DETR pioneers anchor-free, NMS-free end-to-end detection via global self-attention. Co-DETR optimizes encoder feature learning, and DDQ enhances efficiency through dense query selection. For real-time detection, RT-DETR adopts a hybrid CNN-Transformer encoder architecture, and RF-DETR optimizes the network via neural architecture search. For remote sensing-specific tasks, MSRS-DETR [19] designs frequency-aware backbones and bidirectional shallow feature enhancement, and M2S-DETR [20] constructs mixed receptive fields and multi-positional encoding for SAR ship detection. Recent works including CSFPR-RTDETR [21] fuse spatial-frequency features to reduce parameters by 29.1% for UAV small-object detection, WRRT-DETR [22] designs gated global-local attention and attention-guided cross-fusion for maritime scenes under harsh weather, and QEDetr [23] proposes rotation-aligned deformable attention and multi-scale bilinear fusion for fine-grained oriented remote sensing targets. FSDETR [24] builds a frequency-spatial feature enhancement framework for small-object detection via spatial hierarchical attention and frequency-spatial feature pyramids, but it does not optimize for the unique edge characteristics of remote sensing ships. Therefore, encoder feature utilization can be further optimized in existing RT-DETR-s variants for maritime remote sensing, which motivates our encoder enhancement strategy.

### 2.2. Encoder Feature Enhancement Mechanisms

Attention mechanisms adaptively adjust feature responses to focus on salient regions. Channel attention modules such as SE-Net [25] and ECA-Net [26] recalibrate features by modeling inter-channel dependencies, while spatial attention CBAM [27] generates spatial weight maps via pooling and convolution. Self-attention enables global QKV interaction yet suffers from quadratic computational complexity, limiting its direct deployment on high-resolution feature maps. To tackle this, Swin Transformer [28] uses window-localized self-attention, PVT [29] reduces key-value dimensions via spatial reduction, SHViT [30] proposes Single-Head Self-Attention (SHSA) with channel splitting and minimal projection, and TinyNeXt [31] further optimizes projection efficiency via LeanSHSA.

Studies integrating edge cues into attention remain scarce. EGSA [32] realizes multi-task fusion via edge-guided spatial attention, AUHF-DETR [33] partitions spatial attention to reduce Transformer complexity for embedded devices, and BAFNet [34] designs a foreground-background dual-stream attention module via semantic-guided spatial separation for small-object enhancement. However, all the above methods apply edge modulation after attention score calculation, rather than embedding edge priors into the generation of attention weights. Our EASA directly injects edge bias into softmax, achieving deep integration of edge cues and self-attention.

Feature Pyramid Network (FPN) [35] propagates deep semantic features top-down for multi-scale detection, followed by PANet [36] with bottom-up aggregation and BiFPN [37] with learnable fusion weights. LDA-DETR [38] adopts lightweight dynamic attention for small-object detection. Injecting shallow information into deep layers to compensate for small-object information loss has attracted growing interest. Association Encoder [39] extracts background context from shallow layers for deep injection, and FM-RTDETR [40] builds linear-complexity multi-scale aggregation via Mamba state-space models. Inspired by these works, we design the SGDF module, which conducts attention enhancement on full-resolution shallow features before downsampling and cross-scale fusion.

### 2.3. Bounding Box Regression Loss Functions

Bounding box loss directly governs localization quality. The original IoU metric yields zero gradients for non-overlapping boxes. GIoU [41] introduces a minimum enclosing rectangle penalty to address this issue. DIoU [42] adds center distance regularization for faster convergence, CIoU [43] further incorporates aspect ratio consistency, and EIoU [44] decouples width and height penalties. SIoU [45] introduces angular loss, and WIoU [46] proposes outlier-aware dynamic focusing; its v3 variant features non-monotonic gradient adjustment for hard samples. MPDIoU [47] replaces aspect ratio loss with corner distance constraints. This paper systematically compares GIoU, DIoU, CIoU, EIoU, SIoU, WIoUv3 and MPDIoU, ultimately selecting WIoUv3 as the regression loss.

## 3. Methods

### 3.1. Overall Architecture

**Figure 1:**
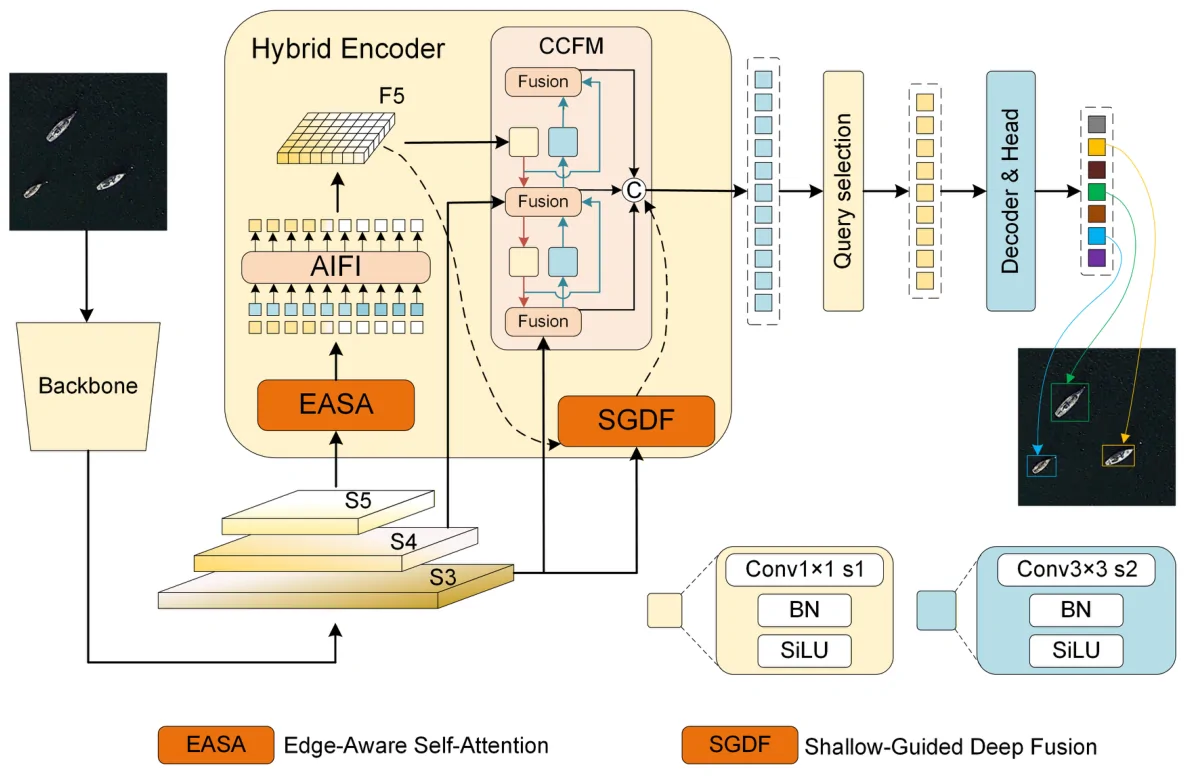
The complete improved model architecture. Modules highlighted in red (EASA and SGDF) denote the proposed improvements; other modules belong to the baseline RT-DETR-s.

We build our improved model based on the RT-DETR-s baseline while retaining its original overall pipeline. As illustrated in Figure 1, the HGNetv2 backbone network is adopted to extract multi-scale feature maps S3, S4 and S5 with strides of 8, 16 and 32, respectively. Two customized improvements are embedded inside the hybrid encoder:

1. The EASA module is inserted before AIFI to perform edge-guided global context enhancement on the deep features projected from S5;
2. After the output of CCFM, the shallow feature S3 from the backbone is fed into the deepest output layer via the SGDF module, which compensates for the information loss of small ships in deep representations through attention modulation.

Finally, the Query Selection and Decoder & Head modules conduct classification and bounding box regression to generate detection outputs. The original GIoU loss is replaced with WIoUv3 loss for bounding box regression.

### 3.2. Edge-Aware Self-Attention (EASA)

**Figure 2:**
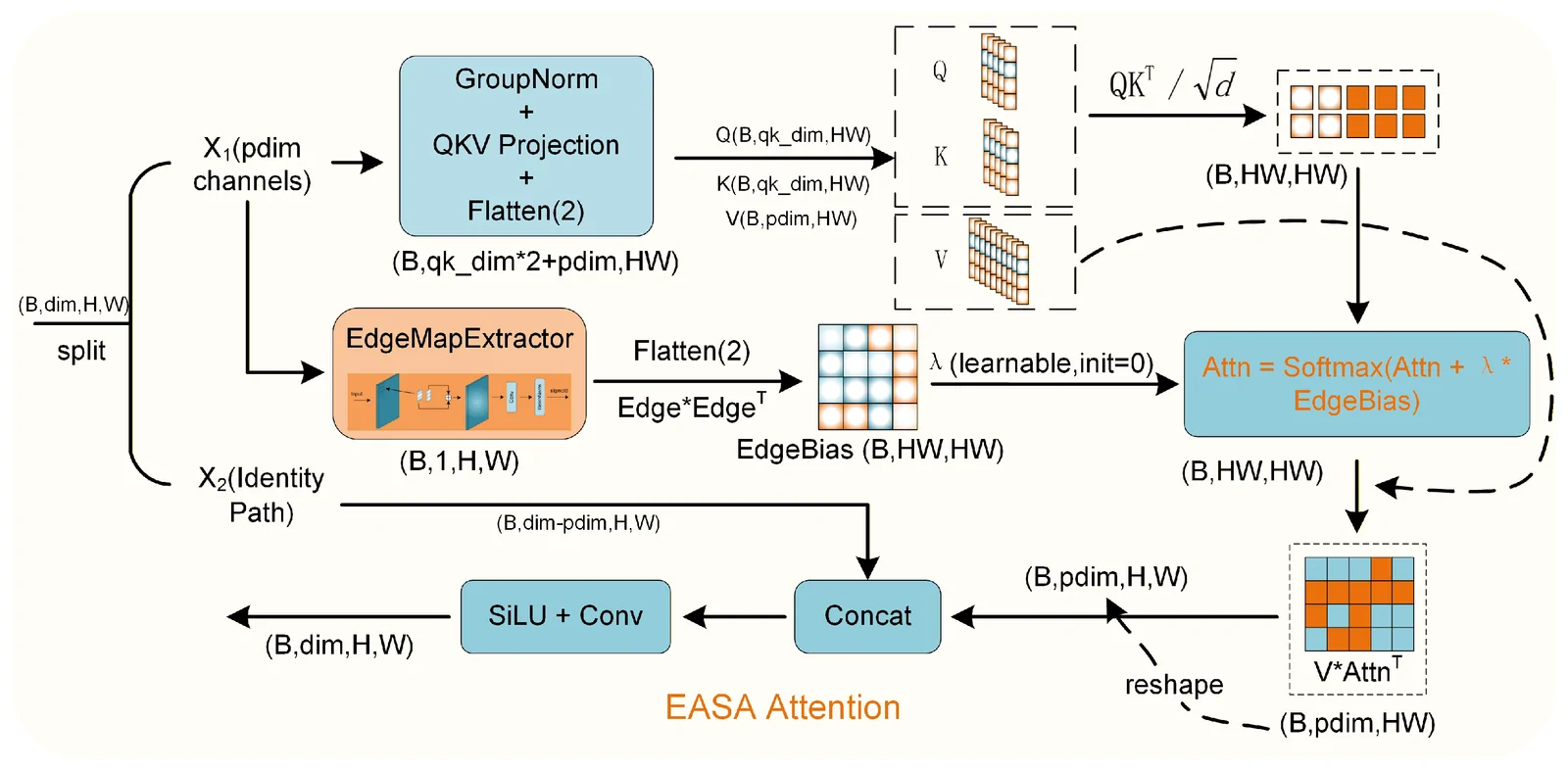
EASA Attention Pipeline. Modules in orange denote the proposed edge-aware components (EdgeMapExtractor and edge bias injection); modules in light blue denote the baseline operations. The symbol “*” denotes matrix multiplication.

**Figure 3:**
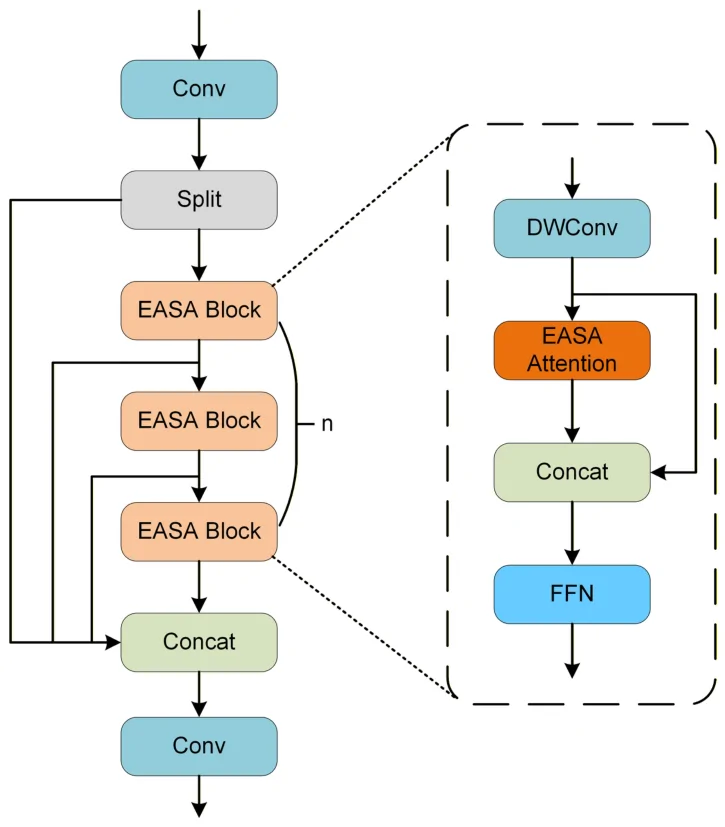
EASA Block Structure.

The AIFI module of vanilla RT-DETR adopts standard multi-head self-attention for global interaction on S5 features, computing attention weights purely from QK semantic similarity without spatial structural cues. In remote sensing ship imagery, ship edges carry critical localization signals, yet semantic similarity fails to distinguish vessel contours from flat ocean backgrounds; the vast ocean region consumes computational resources during global attention and introduces irrelevant noise. Our EASA embeds structural edge priors directly into attention scores before softmax. Unlike post-hoc gating modulation, edge information is internalized into core attention computation. The EASA attention workflow and module structure are shown in Figure 2 and Figure 3, respectively. Given input feature $x\in R^{B\times C\times H\times W}$, we split it along the channel dimension into two branches: $x_{1}\in R^{B\times C_{p}\times H\times W}$ for attention calculation, and $x_{2}\in R^{B\times \left(C-C_{p}\right)\times H\times W}$ as an identity shortcut, where $C_{p}$ defaults to 64. $x_{1}$ sequentially undergoes GroupNorm, 1 × 1 QKV projection and flattening to obtain query $Q\in R^{B\times d_{q}\times HW}$, key $K\in R^{B\times d_{q}\times HW}$ and value $V\in R^{B\times C_{p}\times HW}$ ($d_{q}=16$ by default). Base attention scores are calculated as:

(1) $${\mathrm{Attn}}_{\mathrm{base}}=\frac{Q^{⊤}K}{\sqrt{d}}\in R^{B\times HW\times HW}$$

Meanwhile, an EdgeMapExtractor processes $x_{1}$ to generate edge map $E\in R^{B\times 1\times H\times W}$. The extractor uses fixed, non-learnable Scharr operators to compute gradients, followed by a 1 × 1 convolution, BN and Sigmoid to produce normalized edge intensity maps within [0, 1]. The edge map is flattened into vector $e\in R^{B\times HW\times 1}$, and an outer product constructs the edge bias matrix:

(2) $$\mathrm{Edgebias}=e\cdot e^{⊤}\in R^{B\times HW\times HW}$$

$Edgebias\left[i,j\right]=e_{i}\cdot e_{j}$, which yields maximum values when both positions i,j lie on edges and minimal values for two background pixels. This rank-one construction provides a structural prior that increases attention interactions between any positions with strong edge responses—it does not distinguish between ship contours, coastlines, waves, or wakes. The semantic discrimination of edge types is delegated to the learned query-key projection Attnbase, which encodes semantic similarity. The role of Edgebias is to amplify attention along boundary regions where localization precision is critical; the final attention distribution is determined by the combination Attnbase + λ · Edgebias, where the learned λ controls the strength of the structural prior relative to the semantic attention. The edge bias is injected into attention scores via a learnable scalar λ:

(3) $$\mathrm{Attn}=\mathrm{softmax}\left({\mathrm{Attn}}_{\mathrm{base}}+\lambda \cdot {\mathrm{Edge}}_{\mathrm{bias}}\right)$$

**Figure 4:**
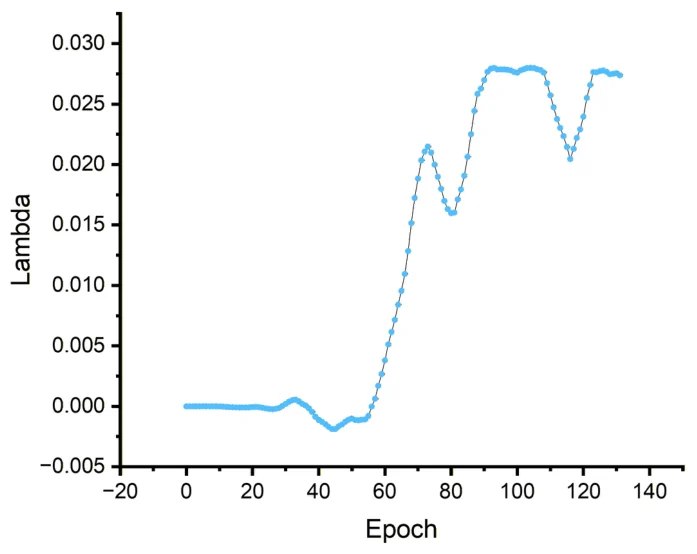
Convergence Curve of λ.

λ is initialized to 0. In early training epochs, EASA degenerates into standard self-attention to avoid interference from edge priors; as training proceeds, the model learns an optimal edge bias intensity. Figure 4 plots the convergence curve of λ. It fluctuates near zero for the first 40 epochs, rises rapidly afterward, peaks at 0.021 around epoch 70, and converges to approximately 0.027 after oscillating between 0.015 and 0.028. The positive converged λ amplifies attention interactions between all positions with strong edge responses (ship contours, coastlines, wakes, etc.), providing a structural prior that complements the semantic selectivity of the learned query-key attention. The combined effect enables the model to prioritize ship-relevant boundary regions where edge structure and semantic cues jointly indicate ship presence. To further quantify the numerical contribution of the edge bias term, we report the statistics of the attention logits after convergence. The semantic attention logits $\frac{Q^{⊤}K}{\sqrt{d}}$ have a mean of 0.0415, standard deviation of 1.5749, and range of [−49.84, 32.50], while the edgebias term λ·Edgebias has a mean of 0.0070, standard deviation of 0.0038, and range of [0.000, 0.026]. The edge bias term constitutes approximately 0.78% of the semantic attention logits in magnitude, indicating that it acts as a gentle structural perturbation that slightly shifts attention toward boundary regions rather than a dominant signal, thereby providing complementary enhancement to semantic attention. The final attention output is obtained by weighting values with attention matrices:

(4) $$x_{1}^{\mathrm{Attn}}=V\cdot {\mathrm{Attn}}^{⊤}\in R^{B\times C_{p}\times HW}$$

After reshaping to restore spatial dimensions, $x_{1}^{Attn}$ is concatenated with the identity branch $x_{2}$, then passed through SiLU activation and a 1 × 1 convolution to project back to the original channel count C.

Table 1 compares SHAB, GatedSHSA and the proposed EASA to validate module effectiveness. SHAB (Spatial Hybrid Attention Block) replaces the original multi-head self-attention in AIFI with a simplified single-head formulation to reduce computational cost while preserving global context modeling. GatedSHSA (Gated Spatial Hybrid Self-Attention) builds upon SHAB by adding a post-hoc gating mechanism: a sigmoid-gated mask is applied to the attention output to filter irrelevant attention responses before feature aggregation. SHAB adopts single-head self-attention with AP50 = 0.841 and APs = 0.229. Building on SHAB, GatedSHSA adds a post-hoc sigmoid gating to the attention output, lifting AP50 by 0.4% and APs by 1.1%, which proves gating’s capacity to filter irrelevant attention responses. Our EASA embeds edge cues directly into softmax scores, boosting AP50 by 0.9% over SHAB and 0.5% over GatedSHSA, with APs improved by 1.4% and 0.3%, respectively, and mAP50–95 reaching 0.597. While the overall mAP50–95 gain over SHAB and GatedSHSA is 0.3%, the improvement is concentrated in the small-object regime (APs +1.4% over SHAB, from 0.229 to 0.243; +0.3% over GatedSHSA, from 0.240 to 0.243). This targeted gain in boundary localization—rather than a uniform increase across all scales—is the expected behavior of an edge-aware mechanism, as edge priors primarily benefit small targets where boundary precision critically affects IoU. Notably, both SHAB and GatedSHSA yield the same mAP50–95 = 0.594, indicating that post-hoc gating alone cannot improve overall detection; it is only when edge cues are integrated into the core attention computation (EASA) that the overall mAP50–95 improves to 0.597.

**Figure 5:**
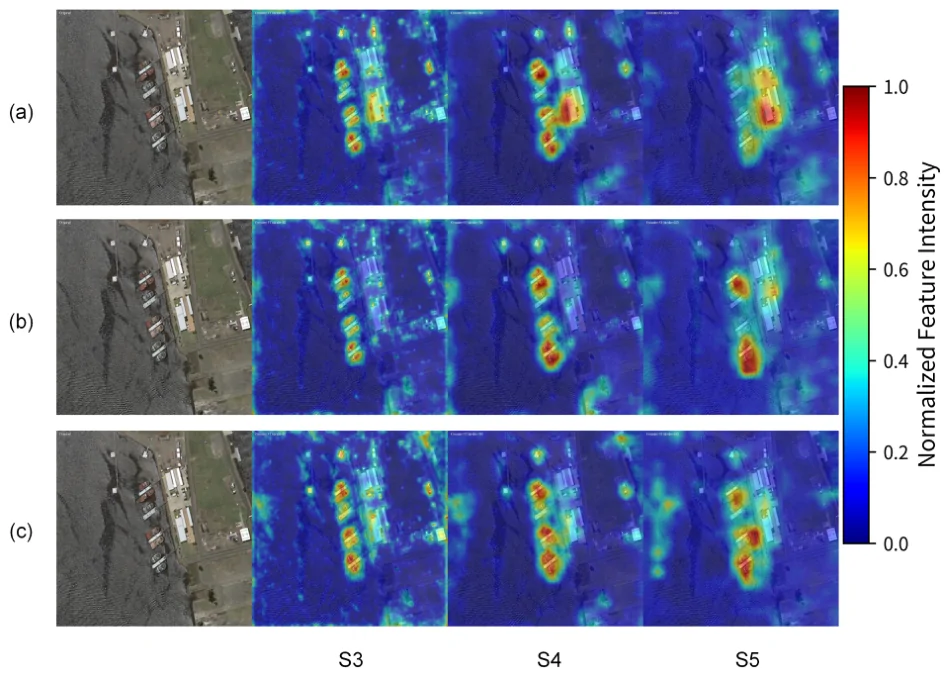
Comparison of attention heatmaps among baseline, SHAB, and EASA (Scene 1): (**a**) baseline model attention map; (**b**) SHAB attention map; (**c**) EASA attention map.

**Figure 6:**
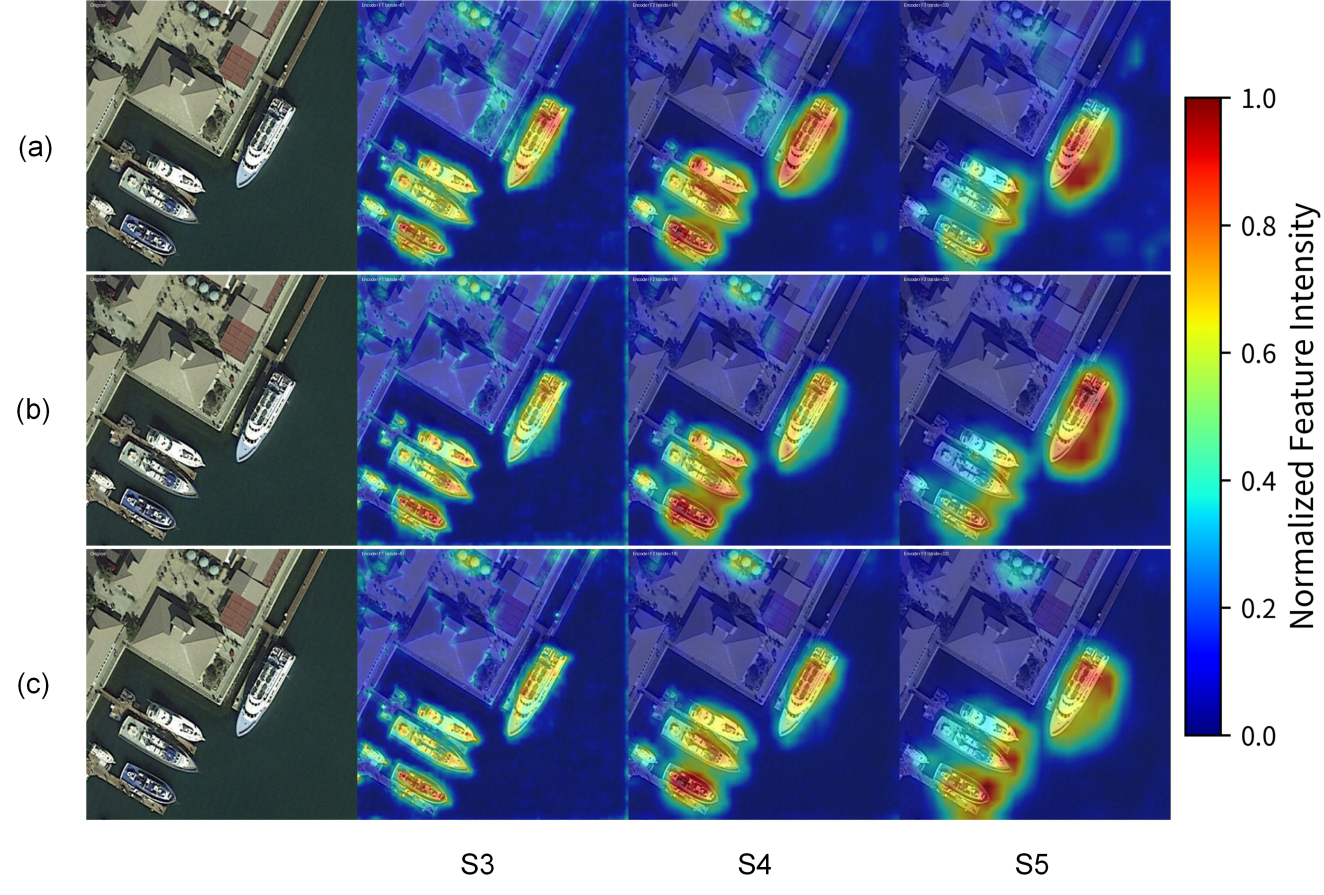
Comparison of attention heatmaps among baseline, SHAB, and EASA (Scene 2): (**a**) baseline model attention map; (**b**) SHAB attention map; (**c**) EASA attention map.

The heatmaps visualize channel-averaged feature maps at each backbone stage. For each feature map $F\in R^{B\times C\times H\times W}$, we compute the spatial mean across the channel dimension: $H\left(i,j\right)={\mathrm{mean}}_{c}\;F\left[b,c,i,j\right]$. All values are min-max normalized to [0,1] with a shared “jet” colormap. A common color bar is included in Figure 5 and Figure 6 to enable quantitative visual comparison across all models and feature levels.

S3 and S4 heatmaps represent backbone outputs before encoder processing. Although EASA directly modifies only S5 features within the hybrid encoder, the observed differences in S3 and S4 maps across models reflect indirect backbone adaptation through end-to-end gradient propagation. During training, the loss gradients flow through the modified S5 pathway back to earlier backbone stages, causing the backbone to adjust its shallow feature representations to better serve the downstream EASA-enhanced encoder. This indirect adaptation is a known phenomenon in end-to-end detection frameworks.

Figure 5 and Figure 6 visualize attention heatmaps of the baseline, SHAB and EASA on two remote sensing scenes. Row (a) denotes baseline RT-DETR, row (b) SHAB, row (c) EASA, with columns corresponding to S3, S4, S5 feature layers. The vanilla baseline disperses attention heavily over ocean wave backgrounds with low weights on ship regions. SHAB slightly converges attention yet fails to distinguish vessel edges from flat water surfaces. EASA suppresses background responses and concentrates attention on ship outlines. We note that this effect is not achieved by the edge bias alone—the rank-one edge bias amplifies attention to all edge regions, including coastlines and wakes. Rather, the improvement results from the combined effect of the learned semantic attention (which distinguishes ship from non-ship edges) and the structural edge prior (which amplifies boundary attention), demonstrating that integrating structural priors into the core attention computation enables more effective boundary localization than post-hoc modulation.

### 3.3. Shallow-Guided Deep Fusion (SGDF)

**Figure 7:**
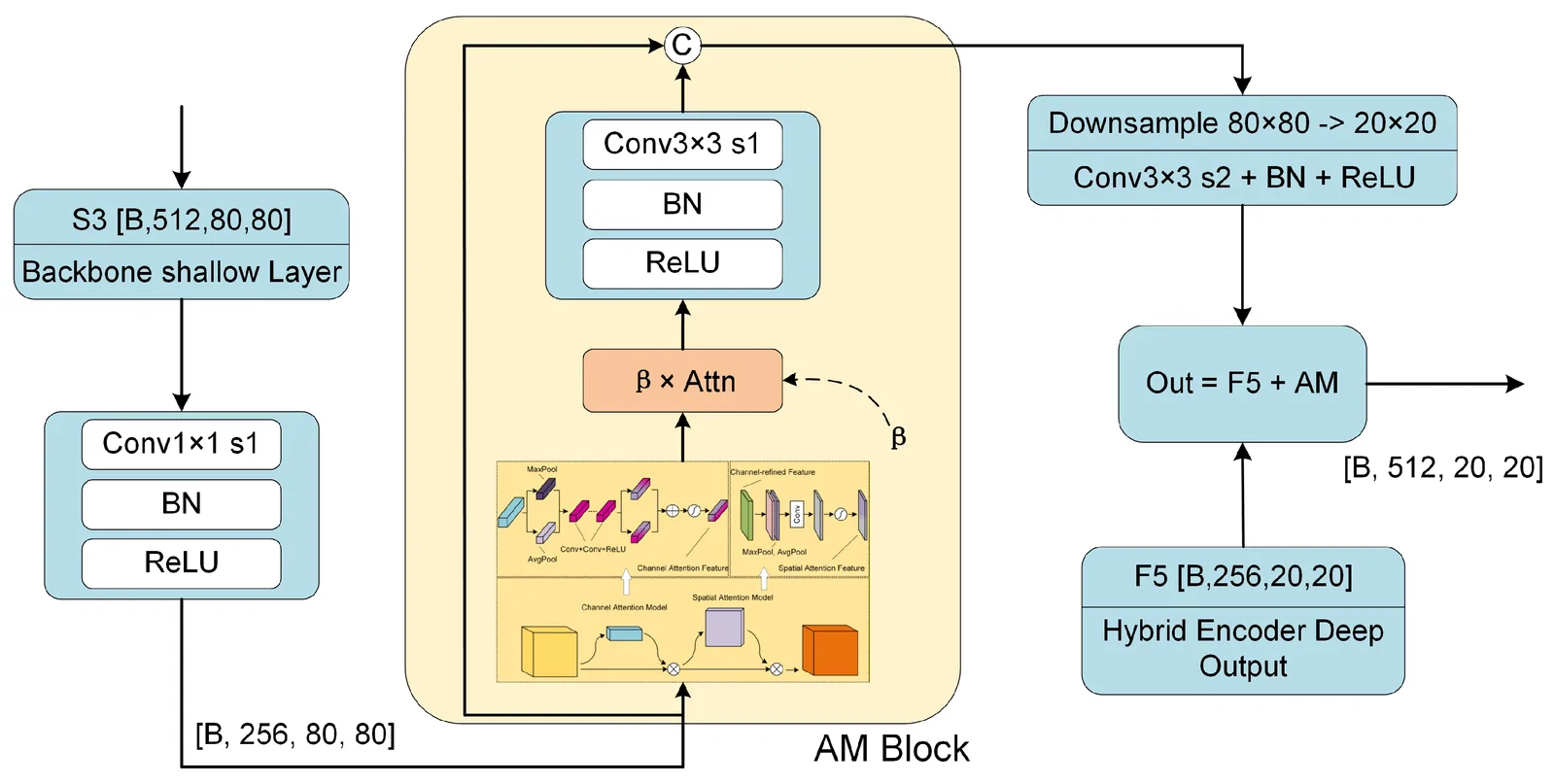
Architecture of the SGDF module. Modules in orange denote the proposed attention-enhanced components (AM Block and learnable scaling factor β); modules in light blue denote standard convolution and fusion operations.

In RT-DETR’s hybrid encoder, S5 features contain rich semantic information but low resolution (20 × 20 for 640 × 640 input). Small ships may occupy merely 1–2 pixels on S5, resulting in severe spatial information loss. By contrast, shallow S3 (stride = 8, resolution 80 × 80) retains fine spatial details critical for small-object localization. We propose the SGDF module following an “attention enhancement at full resolution, then downsampling” paradigm, as shown in Figure 7. While the individual components (CBAM attention and cross-scale fusion) are established techniques, the novelty of SGDF lies in the specific pipeline design: attention enhancement is applied at the original 80 × 80 resolution before downsampling, rather than downsampling first and then applying attention at the reduced resolution, thereby preserving fine-grained spatial detail critical for small-object detection. First, S3 shallow features undergo 1 × 1 convolution, BN and ReLU for channel projection to the hidden dimension. The transformed features are fed into an Attention Module (AM Block) to extract joint channel-spatial attention at the original 80 × 80 resolution. Two consecutive stride-2 3 × 3 convolutions downsample attention-enhanced shallow features to match the 20 × 20 resolution of deep F5 features, which are finally fused via element-wise addition. The internal AM Block adopts CBAM, controlled by a learnable balance coefficient β.

**Figure 8:**
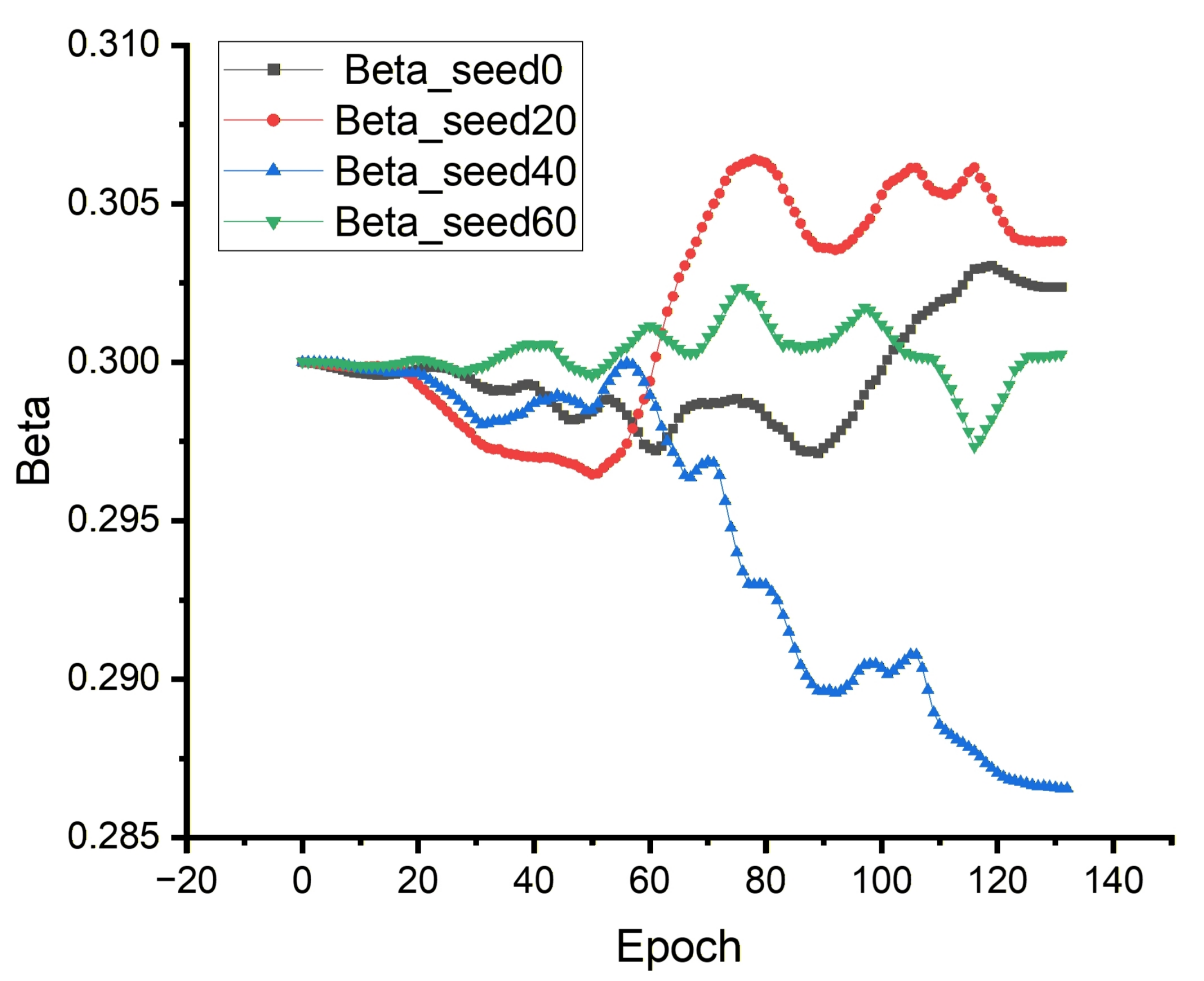
Variation curve of learnable coefficient β.

Figure 8 presents the convergence curves of β across four independent training runs with different random seeds. Initialized at 0.300, β fluctuates within a narrow range and converges to a mean of 0.298 ± 0.008 (range: 0.287–0.304) across the four runs, indicating that the modulation strength remains close to its initialization value. This near-initial convergence suggests that the default modulation strength is approximately appropriate for the shallow-to-deep feature injection task, and the model does not need to substantially deviate from the initial β setting to achieve effective fusion of fine-grained spatial information into deep semantic layers.

### 3.4. Bounding Box Regression Loss

Vanilla RT-DETR uses GIoU loss, which applies a uniform penalty to all samples without accounting for sample quality disparities. We systematically benchmark GIoU, DIoU, CIoU, EIoU, SIoU, WIoUv3 and MPDIoU, selecting WIoUv3 as the final regression loss.

Table 2 presents the comparative experimental results of seven IoU-based loss functions on the remote sensing ship dataset. Taking GIoU as the baseline with AP50 = 0.833 and mAP50–95 = 0.585, DIoU achieves the highest AP50 of 0.858, corresponding to a 2.5% improvement, yet it only raises APs by 0.1%, delivering limited gains for small ships. WIoUv3 attains balanced overall performance, with AP50 = 0.847 (+1.4%), AP75 = 0.644 (+1.5% against the baseline), APs = 0.237 (+1.0%) and mAP50–95 = 0.599 (+1.4%). Although SIoU obtains an AP50 of 0.855 (+2.2%), its APl drops to 0.884, which impairs detection performance for large vessels. All metrics of MPDIoU are inferior to the baseline, making it unsuitable for this task. Balancing localization accuracy, small-object detection capability and performance stability for large targets, the non-monotonic focusing mechanism of WIoUv3 adaptively adjusts gradient weights for samples of varying quality and yields the most balanced performance gain in remote sensing ship detection. Accordingly, we adopt WIoUv3 as the bounding box regression loss function in this work.

This paper acknowledges that the selection of WIoUv3 is based on validation performance, which may introduce potential model selection bias. To address this concern, this paper conducts an independent evaluation on the Ships in Aerial Images test set (1573 images), a benchmark not used during training or model selection. All seven IoU loss variants (GIoU, CIoU, DIoU, EIoU, SIoU, MPDIoU, and WIoUv3) are trained on the primary Ship-Detection dataset and evaluated on this unseen test set without any fine-tuning. As shown in Table 3, WIoUv3 achieves the best mAP50–95 of 0.090, outperforming the GIoU baseline (0.087) by 0.003 and CIoU (0.079) by 0.011. The advantage is most pronounced in medium object detection (APm = 0.176 vs. 0.152 for GIoU), representing an improvement of 0.024. These results confirm that the selection of WIoUv3 generalizes to unseen data distributions and is not biased toward the validation set. Additionally, the cross-dataset generalization experiment (Section 4.5) further validates this selection on the validation split of the same independent dataset.

## 4. Experiments and Analysis

### 4.1. Datasets and Experimental Setup

All experiments adopt unified hardware and software configurations for reproducibility, listed in Table 4. Two publicly available optical remote sensing ship detection benchmarks are used in this work. The primary dataset (referred to as Ship-Detection [48], available on Kaggle) is used for ablation, comparison, and stability experiments. It contains 496 training images and 125 validation images at a resolution of 400×360 pixels, with a total of 1705 annotated ship instances in the training set and 246 in the validation set (1951 in total). The object-size distribution (following the COCO area-based criterion) is as follows: 66.2% of ships are small (area ≤32×32 pixels), 25.0% are medium (32×32≤ area ≤96×96 pixels), and 8.9% are large (area ≥96×96 pixels), making it particularly suitable for evaluating small-object detection performance. No test split is provided; the validation set is used for all reported metrics.

The secondary dataset (referred to as Remote-Ship [49], also available on Kaggle) is used for generalization evaluation. It contains 3300 training images and 412 validation images at a resolution of 600×600 pixels, with 10,485 and 1207 annotated ship instances, respectively (11,692 in total). This dataset features more complex scenes including dense harbors, coastline clutter, and broader-scale distributions: 55.6% small, 24.6% medium, and 19.8% large.

Standard preprocessing includes resizing all images to 640 × 640 and ImageNet normalization. During training, the RT-DETR-s default data augmentation pipeline is applied, including Mosaic, HSV color-space augmentation, random horizontal flip, and multi-scale random resize. No additional preprocessing or exclusion criteria were applied beyond the model’s default settings. All compared models are trained using the same dataset splits, identical data augmentation strategies (mosaic, HSV color-space augmentation, random horizontal flip, and multi-scale random resize), the same training budget (132 epochs for the primary dataset and the secondary dataset), 640 × 640 input resolution, and the same model selection criterion (best validation mAP50–95). All models use their officially released configurations and pretrained backbone weights. Both datasets follow the COCO annotation format with a single ship category.

To evaluate the generalization capability of the proposed model, we introduce an additional public dataset, Ships in Aerial Images, as an independent test set. This dataset is publicly available on the Kaggle platform and contains aerial imagery of maritime vessels captured from various altitudes and viewing angles. The dataset comprises a total of 13,435 images divided into three subsets: 9697 training images, 2165 validation images, and 1573 test images, with 23,176 annotated ship instances in the training and validation sets combined. A distinctive characteristic of this dataset is the high proportion of small objects. Small targets account for 58.8% of all annotations, medium targets for 22.7%, and large targets for 18.5%. This size distribution differs significantly from the primary training dataset, presenting a substantial domain shift challenge. The prevalence of small, low-resolution ship instances under varying aerial perspectives makes this dataset particularly suitable for assessing cross-dataset generalization.

We note that the primary dataset does not include a separate test split; following standard COCO benchmarking practice, model selection is based on the best mAP50–95 on the validation set. To mitigate potential model-selection bias, the secondary dataset (Remote-Ship) provides an independent evaluation benchmark with entirely disjoint training and validation partitions, ensuring that the final performance gains are not artifacts of validation-set overfitting.

### 4.2. Evaluation Metrics

We follow the MS COCO evaluation protocol. Precision metrics include mAP50–95, AP50 and AP75 calculated at IoU thresholds of 0.5 and 0.75, respectively. Scale-specific metrics APs (small), APm (medium), APl (large) measure performance across object sizes. Inference speed is evaluated via FPS; 30 FPS is regarded as the minimum threshold for real-time applications. FPS is measured under the following protocol: numerical precision is FP32; warm-up consists of 100 iterations; inference is averaged over 300 repetitions; batch size is 1; pre-processing (resize and normalization) and post-processing (sigmoid activation and confidence filtering) are included; CUDA synchronization is enabled; and a 5% trimmed mean is applied to remove outliers. All models are evaluated on the same hardware (NVIDIA RTX 3090D GPU) under identical conditions.

### 4.3. Comparative Experiments

**Figure 9:**
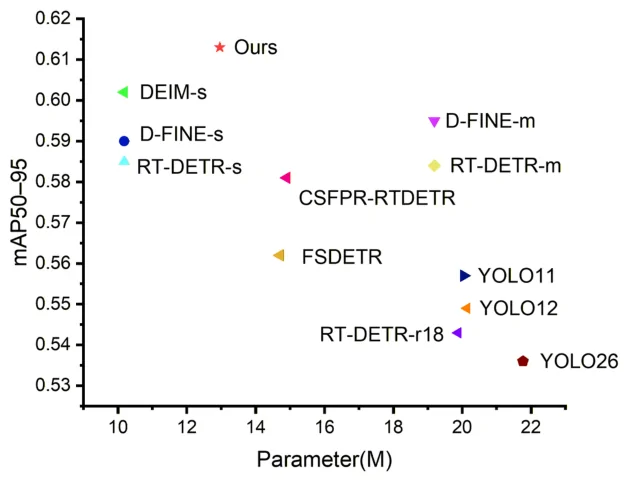
Scatter plot of mAP50–95 versus parameter count for all compared models.

Table 5 reports quantitative comparisons between our method and eleven mainstream detectors, including recently proposed small-object detection methods CSFPR-RTDETR [21] and FSDETR [24] with advanced frequency-spatial feature enhancement mechanisms. Figure 9 plots the mAP50–95 vs. Parameter count trade-off curve. Our model outperforms all competitors across all precision metrics, reaching AP50 = 0.858 and mAP50–95 = 0.613, corresponding to 2.5% and 2.8% gains over baseline RT-DETR-s. APs increases from 0.224 to 0.256 (+3.2%), confirming substantial improvements for small ships. Compared with recent feature-enhanced DETR variants, FSDETR achieves mAP50–95 = 0.562 with 14.7 M parameters, while CSFPR-RTDETR attains mAP50–95 = 0.581 with 14.9 M parameters and 63.9 GFLOPs. Our method outperforms both: +5.1% mAP50–95 and +8.9% APs over FSDETR with fewer parameters; +3.2% mAP50–95 and +7.1% APs over CSFPR-RTDETR with 13.0% fewer parameters and 32.0% fewer GFLOPs, achieving a better accuracy-efficiency trade-off. Among DETR variants, DEIM-s achieves mAP50–95 = 0.602, 1.1% lower than ours. D-FINE-m with 19.19 M parameters only reaches mAP50–95 = 0.595; our model attains higher accuracy with merely 67.5% of its parameter budget (12.96 M). Compared with RT-DETR-m (19.19 M), our lightweight architecture delivers superior precision with fewer parameters.

YOLO11 and YOLO26 achieve high FPS (106.8 and 106.4) yet low AP50 (0.793 and 0.794) and roughly 1.5× larger parameter sizes. YOLO12 attains AP50 = 0.815, still 4.3% lower than the proposed method. D-FINE-s performs comparably to RT-DETR-s but underperforms our model by 5.2% on APs. RT-DETR-r18 yields the lowest AP50 of 0.785 among all DETR detectors. Overall, our framework achieves state-of-the-art precision among DETR-based detectors with 37.6 FPS, 12.96 M parameters and 43.42 GFLOPs, with dominant advantages in small-object detection.

The proposed model increases GFLOPs from 24.82 to 43.42 (a 75% increase), primarily due to the SGDF module’s attention computation on high-resolution (80 × 80) shallow features. While this represents a significant theoretical computation increase, the actual inference speed decreases only from 40.9 to 37.6 FPS (an 8.1% reduction), as the hybrid encoder’s AIFI and CCFM dominate the inference latency. The model remains well above the 30 FPS real-time threshold.

### 4.4. Ablation Experiments

As shown in Table 6, this paper conducts ablation experiments comparing three edge extraction strategies: the fixed Scharr operator (our default), the Sobel operator, and a learnable Scharr operator (initialized with Scharr weights but allowing gradient updates during training). All three variants share identical model architecture, differing only in the edge extraction module, ensuring a fair comparison. The fixed Scharr operator achieves the best overall performance with mAP50–95 of 0.597, outperforming the Sobel operator by 0.008 and the learnable Scharr by 0.021. The advantage is most pronounced in small object detection (APs), where Scharr reaches 0.243 compared to 0.195 (Sobel) and 0.209 (Learnable-Scharr), representing improvements of 0.048 and 0.034, respectively. This validates the design choice: the Scharr operator’s higher rotational symmetry and more accurate gradient approximation provide superior edge responses for ship boundary localization, particularly for small targets where precise edge information is critical.

The learnable Scharr operator underperforms both fixed variants, achieving mAP50–95 of only 0.576. This suggests that allowing the edge extractor to update freely introduces optimization difficulty: the network may shift the filter toward task-specific features that deviate from the fundamental edge-detection prior, leading to overfitting on training data characteristics. The Sobel operator achieves competitive performance on medium and large objects (APm = 0.779, APl = 0.926), but its weaker small-object performance (APs = 0.195) confirms that its lower gradient accuracy compared to Scharr is a limiting factor. These results demonstrate that a well-designed fixed operator (Scharr) provides a better inductive bias for ship detection than both a weaker fixed operator (Sobel) and a learnable alternative, justifying the use of the fixed Scharr operator in the final model.

Table 7 reports ablation results with RT-DETR-s as the baseline (mAP50–95 = 0.585, APs = 0.224). Adding standalone EASA lifts mAP50–95 by 1.2% and APs by 1.9%, with FPS only dropping from 40.9 to 38.7 and parameter overhead limited to 0.34 M, demonstrating a high precision-efficiency trade-off. Independent SGDF injection increases mAP50–95 by 1.5% and APs by 1.1%, yet raises GFLOPs substantially from 24.82 to 43.14. Simply replacing GIoU with WIoUv3 improves AP75 by 1.5% without any extra parameters or latency overhead, even slightly boosting FPS to 41.5.

Dual-module combinations show complementary effects: EASA + SGDF yields mAP50–95 = 0.606 (+2.1%); SGDF + WIoUv3 achieves the highest AP75 at 0.655, though this marginally surpasses the full model (0.651) within training variance; EASA + WIoUv3 achieves competitive accuracy with minimal parameters (10.52 M) and FPS = 39.7. Integrating all three components (full model) delivers optimal performance across key metrics: AP50 = 0.858 (+2.5%), APs = 0.256 (+3.2%), mAP50–95 = 0.613 (+2.8%) at 37.6 FPS and 12.96 M parameters. The significant APs gain of 3.2% validates strong synergy between edge-aware attention and shallow cross-scale fusion: EASA enhances boundary localization while SGDF recovers lost fine-grained spatial information, with WIoUv3 further refining box regression quality.

We selected the Scharr operator for edge extraction due to its higher rotational symmetry and more accurate gradient magnitude compared to Sobel. A fixed (non-learnable) operator was chosen to ensure that the edge prior remains a stable structural signal independent of learned parameters, preventing the edge extractor from degenerating into a feature-dependent filter that could overfit to training data.

To evaluate the sensitivity of detection performance to the edge bias strength λ, this paper fixes λ at six discrete values (0, 0.01, 0.02, 0.03, 0.05, 0.10) and retrains the full model for each setting while keeping all other components identical. As shown in Table 8, setting λ = 0 (disabling the edge bias entirely) yields the worst overall performance (mAP50–95 = 0.573), confirming that the edge-aware modulation is essential. Performance improves steadily as λ increases from 0 to 0.02, peaking at mAP50–95 = 0.593, but begins to degrade beyond 0.03, with λ = 0.05 and λ = 0.10 producing mAP50–95 of 0.581 and 0.584, respectively. This indicates that excessive edge bias overemphasizes high-frequency noise and disrupts the semantic attention distribution. The learnable λ converges to approximately 0.027, which falls within the optimal range (0.02–0.03) and achieves mAP50–95 = 0.597, surpassing all fixed values. Notably, the learnable mechanism provides the largest improvement in small object detection (APs = 0.243 vs. 0.233 for the best fixed λ), demonstrating that adaptive parameter optimization is more effective than manual tuning.

An analogous sensitivity analysis is conducted an analogous sensitivity analysis for the SGDF modulation strength β by fixing it at five discrete values (0.1, 0.2, 0.3, 0.4, 0.5). As shown in Table 9, performance peaks around β = 0.2–0.3 (mAP50–95 = 0.596 and 0.595, respectively) and degrades at both extremes, with β = 0.1 and β = 0.5 yielding mAP50–95 of 0.591 and 0.588. The learnable β converges to approximately 0.298, which aligns almost exactly with the empirical optimum of 0.3, and achieves the best mAP50–95 of 0.600. The improvement is again most pronounced for small objects (APs = 0.235 vs. 0.232 for the best fixed β), confirming that the learnable mechanism effectively identifies the optimal operating point without manual search.

These sensitivity analyses yield two key findings. First, both λ and β exhibit clear performance curves with distinct optimal regions, validating that the learnable mechanism converges to near-optimal values rather than trivial solutions. Second, the learnable parameters consistently outperform all fixed values, particularly in small object detection, which suggests that the adaptive mechanism captures data-dependent dynamics that no single fixed value can encode. The convergence values (λ≈ 0.027, β≈ 0.298) fall squarely within the empirically optimal ranges, demonstrating that the proposed learnable coefficient design is both effective and well-justified.

### 4.5. Generalization Experiments

Generalization evaluation is conducted on the Remote-Ship dataset, which features more complex scenes and broader target scale distributions, as shown in Table 10. All models are trained from scratch on its 3300 training images and evaluated on the 412 validation images. This independent benchmark also serves to verify that the improvements observed on the primary dataset are not due to validation-set overfitting, as all models are retrained from scratch on a completely different dataset. Compared to the primary Ship-Detection dataset, this benchmark introduces denser harbor scenes and more varied coastal backgrounds, posing greater challenges for ship detection. Notably, the Remote-Ship dataset contains images with significant sensor noise, reduced contrast, and complex sea clutter. The competitive performance of the proposed method on this dataset—APs improvement of 2.9% and mAP50–95 improvement of 1.5% over the baseline RT-DETR-s demonstrates that EASA and SGDF maintain effectiveness under degraded imaging conditions, where edge information is inherently noisy and less reliable. While YOLO26 achieves the highest overall mAP50–95 = 0.401, it requires 21.77 M parameters and 75.40 GFLOPs, 1.68× and 1.74× higher than our model, respectively.

Among DETR variants, our method achieves competitive generalization performance with competitive efficiency. Among the compared feature-enhanced variants trained on the same Remote-Ship dataset, FSDETR achieves mAP50–95 = 0.355 and APs = 0.239, while CSFPR-RTDETR attains mAP50–95 = 0.344 and APs = 0.232. Our method outperforms both on mAP50–95 (+1.2% and +2.3%, respectively) and APs (+2.5% and +5.6%, respectively), demonstrating more effective feature enhancement for ship detection under complex remote sensing scenes. Compared with lightweight DETR baselines and feature-enhanced variants, our method improves AP50 by 2.7–3.9% and APs by 2.9–8.9%. Even against heavier RT-DETR-m and D-FINE-m (19.19 M), our model surpasses them on AP50, mAP50–95 and APm with only 67.5% parameter count. RT-DETR-r18 exhibits the weakest generalization performance, with all metrics lagging behind competitors. Notably, YOLO architectures suffer drastic APs degradation: YOLO26 only reaches APs = 0.170, 44.1% lower than our 0.245; YOLO11 attains merely 0.139 (56.7% of our performance). Compared with the RT-DETR-s baseline, our method raises AP50 by 2.7%, mAP50–95 by 1.5% and APs by 2.9% on this more challenging benchmark, demonstrating the effectiveness of EASA and SGDF for tiny ship detection under complex remote sensing scenes, particularly in APs and parameter efficiency, although YOLO26 achieves higher overall mAP50–95 with significantly more parameters. Claims of superiority are restricted to the DETR category and specific metrics (AP50, APs) where our method demonstrates clear advantages.

Table 11 presents the cross-dataset generalization results of twelve detection models on the Ships in Aerial Images test set. All models exhibit significant performance drops compared to their results on the primary dataset, confirming the challenge of cross-dataset generalization. The mAP50–95 values range from 0.099 to 0.117, which are substantially lower than typical in-dataset performance. This degradation is expected because the independent dataset differs in imaging conditions, object scale distributions, and scene complexity, creating a substantial domain gap that models trained solely on the primary dataset cannot fully bridge.

Despite the domain shift, our model achieves the highest mAP50–95 of 0.117, outperforming the second-best models (CSFPR-DETR and DEIM-s, both at 0.112–0.113) by 0.4–0.5%. In AP50, our model reaches 0.241, surpassing the next best (YOLO26 and CSFPR-DETR at 0.230) by 1.1%. This indicates that the Edge-Aware Self-Attention (EASA) and Shallow-Guided Deep Fusion (SGDF) modules learn more transferable features that generalize better to unseen data distributions.

The independent dataset is particularly challenging due to its high proportion of small objects (58.8%). Our model achieves the best APs of 0.025, outperforming all competitors (0.016–0.023) by 0.2–0.9% over the second best. This demonstrates that the fixed Scharr operator in EASA captures fundamental edge features that remain effective even under distribution shift. However, the absolute APs value of 0.025 indicates that small object detection under cross-dataset conditions remains an open challenge, as the edge responses become less reliable on unfamiliar image characteristics.

Notably, our model achieves the best generalization performance while maintaining competitive efficiency. With only 12.96 M parameters and 43.42 GFLOPs, it is significantly lighter than YOLO-series models (20–22 M params, 68–75 GFLOPs) and medium-scale DETR models (19–20 M params, 56–60 GFLOPs), yet outperforms all of them. Compared to small-scale models with similar efficiency (D-FINE-s, DEIM-s, RT-DETR-s at 10 M params, 25 GFLOPs), our model achieves 0.5–1.8% higher mAP50–95, demonstrating that the proposed architecture offers a better generalization-efficiency trade-off.

The results also reveal that all models struggle with small and medium objects under domain shift, with APs values below 0.026 across the board. This suggests that the domain gap primarily affects fine-grained feature extraction for small targets. The fixed Scharr operator, while providing consistent edge priors on the training distribution, may produce suboptimal responses under different imaging conditions, leading to degraded attention guidance. This observation motivates future work on adaptive edge operators or learned edge priors that can dynamically adjust to varying image characteristics.

In summary, the cross-dataset generalization experiment demonstrates that the proposed model achieves superior transferability compared to eleven state-of-the-art detectors while maintaining competitive computational efficiency. The results validate the effectiveness of the EASA and SGDF modules in learning transferable representations, while also highlighting the inherent limitations of fixed edge operators under domain shift.

It should be noted that the loss function comparison (Section 3.4, Table 3) is evaluated on the test split of the Ships in Aerial Images dataset, while the cross-dataset generalization experiment (Table 11) is evaluated on the validation split. This separation ensures that the loss function selection and the generalization assessment are based on non-overlapping data partitions, further strengthening the validity of the evaluation.

### 4.6. Training Stability Analysis

Four training runs with random seeds [0, 20, 40, 60] are conducted to analyze training stability; all other experiments adopt seed = 40. Table 12 presents baseline RT-DETR-s statistics, while Table 13 reports results of our improved model. Baseline RT-DETR-s: mAP50–95 mean = 58.73%, std = 0.311%, range = 0.8%; AP50 mean = 83.65%, std = 0.568%, range = 1.5%; AP75 mean = 63.05%, std = 1.346%, range = 3.8% (largest fluctuation); APs mean = 22.53%, std = 0.487%.

Proposed full model: mAP50–95 mean = 60.98%, std = 0.217%, range = 0.6%; AP50 mean = 85.5%, std = 0.187%, range = 0.5%; AP75 mean = 64.8%, std = 0.178%, range = 0.5%; APs mean = 25.3%, std = 0.274%. All standard deviations are substantially reduced: mAP50–95 std drops by 30.2%, AP50 std by 67.1%, AP75 std by 86.8%, APs std by 43.7%. While the number of repetitions (4 seeds) is limited, the consistent reduction across all metrics suggests that the proposed modules tend to reduce performance variance across random initializations. Furthermore, the four independent training runs under different random seeds provide additional evidence that the observed performance gains are robust to initialization variance rather than artifacts of validation-set overfitting to a single random seed. A more rigorous statistical analysis with additional runs is planned in future work.

### 4.7. Qualitative Visualization

**Figure 10:**
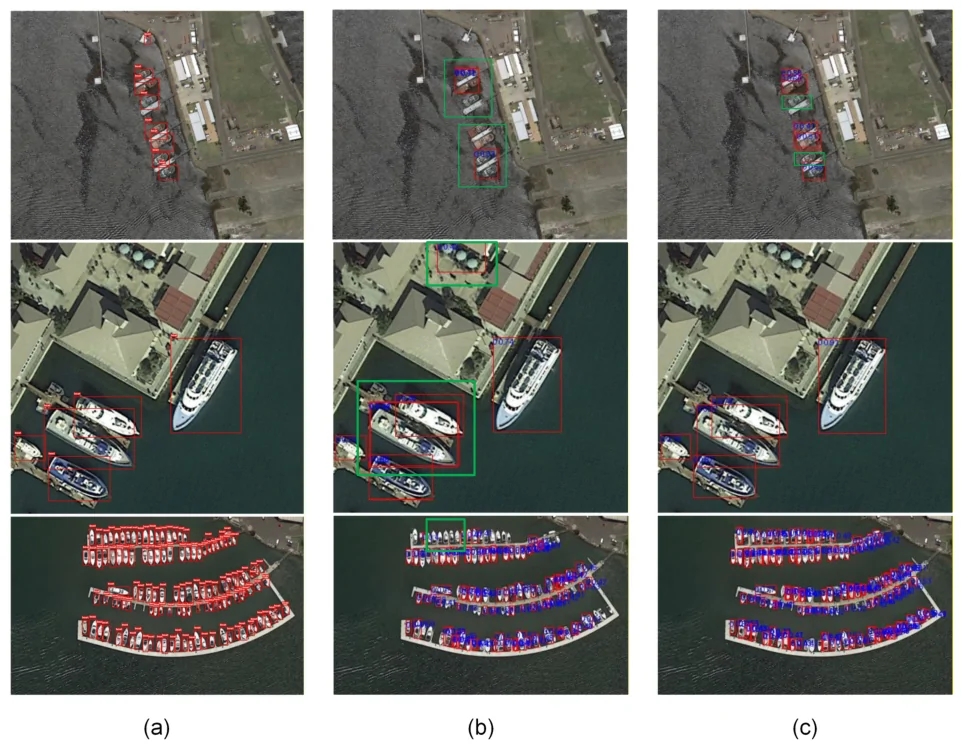
Qualitative comparison on the Ship-Detection dataset. Columns (**a**–**c**) represent ground truth, baseline RT-DETR-s predictions, and our improved model predictions, respectively. Red boxes denote ground truth annotations with class labels, and green boxes indicate missed targets. Rows correspond to sparse, medium-density, and dense harbor scenarios.

**Figure 11:**
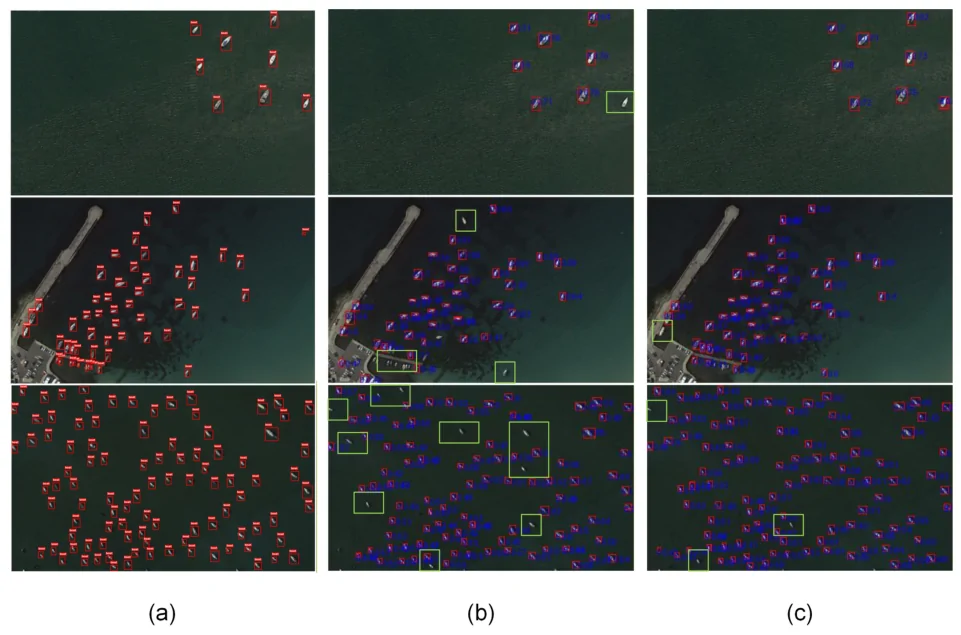
Qualitative comparison on the Remote-Ship dataset. Columns (**a**–**c**) represent ground truth, baseline RT-DETR-s predictions, and our improved model predictions, respectively. Red boxes denote ground truth annotations with class labels, and green boxes indicate missed targets. Rows correspond to sparse, medium-density, and dense harbor scenarios.

Figure 10 and Figure 11 visualize the detection performance of the proposed model from the perspectives of missed detections and false positives via a 3 × 3 grid layout. In each subgraph, column (a) denotes ground-truth annotations, column (b) shows prediction results of the baseline model, and column (c) presents outputs of our improved model. Green bounding boxes are used to mark missed targets, and rows correspond to three scenarios: sparse, medium-density and dense harbors.

As shown in Figure 10, the baseline only detects 2 ships in the sparse scene, while our method successfully identifies all 5 vessels. For medium-density areas, the baseline suffers from false positives and duplicate detections, whereas our model detects all targets with steady confidence scores across all object scales. The performance gap is the most prominent in dense dock scenes, where the baseline generates massive missed detections, and our method achieves a remarkable rise in detection recall.

In Figure 11, the baseline misses a tiny isolated vessel at the bottom-right corner in the sparse scene, which is fully captured by our approach. In medium-density port zones, the baseline fails to detect numerous clustered ships along the coastline, yet our model only misses a single target. The discrepancy becomes even larger under dense conditions: the baseline yields substantial missed vessels at both the center and periphery of crowded regions, while our method only loses a few dim tiny targets at extreme edges. Overall, the proposed approach significantly reduces missed detections in dense scenarios and delivers improved detection capacity for closely packed small ships. This is further supported by the APs improvement on the primary dataset (0.256 vs. 0.224 for the baseline, +3.2%), which serves as a quantitative indicator of enhanced small-object detection capability.

**Figure 12:**
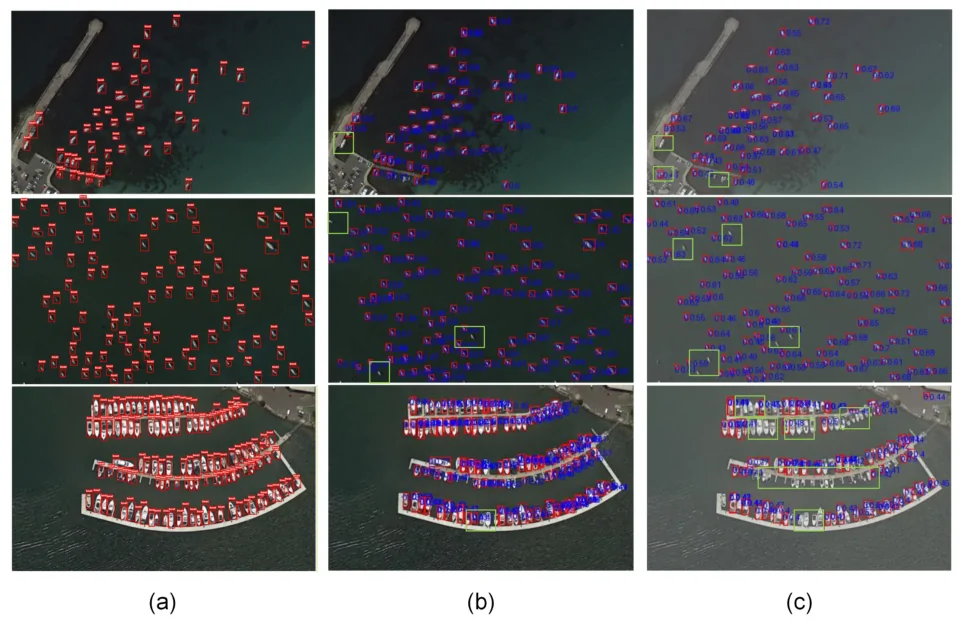
Qualitative comparison of detection results under foggy conditions. Columns (**a**–**c**) represent ground truth annotations, detection results on clear images, and detection results on foggy images, respectively. Red boxes denote ground truth annotations with class labels, and green boxes indicate missed targets. Rows correspond to sparse harbor, medium-density harbor, and dense dock scenarios.

As shown in Figure 12, we provide a visual evaluation of foggy remote-sensing images to demonstrate these limitations. The fixed Scharr operator fails to produce reliable edge responses under low-contrast conditions, leading to significant increases in missed detections. Future work will explore integrating image restoration and domain adaptation techniques to improve robustness under such adverse conditions.

### 4.8. Limitations and Future Work

This work proposes EASA, SGDF and WIoUv3 improvements upon RT-DETR and achieves significant accuracy gains for optical remote sensing ship detection, yet there remains room for further expansion. Combined with existing research trends in real-time remote sensing detection, future research directions are outlined in four dimensions.

First, optimize the adaptability and lightweight design of feature enhancement modules. Current EASA relies on fixed Scharr operators that provide only a generic edge prior without semantic discrimination. The rank-one edge bias cannot distinguish ship contours from coastlines, waves, or wakes; semantic selectivity is entirely delegated to the learned query-key attention. Additionally, the fixed Scharr operator has not been specifically evaluated under extreme low-contrast conditions, complex sea clutter, and varying illumination scenarios. While the edge bias term constitutes only 0.78% of attention logits in magnitude—providing inherent tolerance to edge extraction noise—a systematic robustness analysis under varying imaging conditions is needed. Future work will conduct controlled robustness experiments under synthetic degradation, including noise injection, contrast reduction, and illumination variation, and explore adaptive edge extraction with illumination-aware thresholding to maintain reliable edge responses across diverse maritime environments. Future work will explore learnable, semantically-aware edge extraction that can directly distinguish ship-relevant boundaries from other scene structures, and SGDF introduces non-negligible computational overhead. Future work will design learnable multi-scale edge extraction branches with scene-adaptive λ bias control. Mamba state-space models will replace stacked convolution downsampling in SGDF for linear-complexity cross-scale propagation, supplemented by frequency-domain feature modules to realize joint spatial-frequency enhancement, balancing precision and inference latency.

Second, extend multi-modal and oriented target detection capabilities. This paper only addresses horizontal bounding box detection on single visible-light imagery with constrained applicable scenarios. As shown in Figure 12, we provide visual evaluation on foggy remote-sensing images to demonstrate these limitations. The fixed Scharr operator fails to produce reliable edge responses under low-contrast conditions, leading to significant increases in missed detections. We will integrate angle-aware edge encoding into EASA and develop rotation-adapted WIoUv3 loss for arbitrarily oriented ships. A shared encoder architecture for optical-SAR and visible-infrared fusion will be constructed, combined with AIS trajectory temporal priors to build spatio-temporal joint detection pipelines supporting all-day maritime monitoring.

Third, boost cross-domain generalization and edge deployment compatibility. We note that the primary dataset does not provide a separate test split, and the validation set is used for both model selection and performance reporting. While the independent Remote-Ship benchmark and multi-seed stability experiments mitigate this concern, a dedicated test set on the primary dataset would provide stronger guarantees against model-selection bias. Furthermore, all datasets adopted in this paper are visible-light remote sensing imagery, leaving cross-sensor and cross-ocean generalization potential untapped. A standardized multi-source composite ship dataset covering optical, SAR and infrared modalities will be established. Adversarial domain adaptation will mitigate domain shift between satellite platforms. Lightweight convolution redesign, hierarchical knowledge distillation and hardware-aware INT8 quantization will be deployed for airborne and spaceborne embedded terminals. Masked self-supervised pre-training will reduce labeling costs, paving the way for few-shot and open-vocabulary ship detection.

Fourth, construct an integrated multi-task interpretation framework. Instance segmentation and fine-grained ship classification branches will be appended to the detection backbone to enable joint reasoning of vessel localization, contour segmentation and model identification. A comprehensive maritime remote sensing interpretation system will be developed, integrating target detection, temporal trajectory analysis and marine anomaly early warning.

## 5. Conclusions

Taking RT-DETR as the baseline, this paper addresses small-object sparsity and boundary localization challenges in remote sensing ship detection with three complementary improvements:

1. The Edge-Aware Self-Attention (EASA) module embeds Scharr edge maps as a learnable structural prior before softmax computation, amplifying attention to boundary regions; combined with the learned semantic query-key attention, this enables the model to prioritize ship-relevant edges for improved localization; 2. The Shallow-Guided Deep Fusion (SGDF) module injects full-resolution shallow spatial cues into deep semantic layers via attention-first downsampling to recover lost small-object details; 3. WIoUv3 regression loss leverages non-monotonic sample focusing to dynamically adjust gradient weights for precise bounding box regression.

Quantitative comparisons confirm our model attains AP50 = 0.858 and mAP50–95 = 0.613, corresponding to 2.5% and 2.8% absolute improvements over RT-DETR-s, with APs elevated by 3.2%. Ablation experiments verify independent performance contributions and strong synergy between EASA and SGDF, with their joint use yielding larger small-object gains than any single component or dual-module combination. Generalization Experiments demonstrate state-of-the-art performance across DETR architectures, with our method demonstrating advantages within the lightweight DETR category, particularly in APs and parameter efficiency. Training stability experiments suggest reduced performance variance across random seeds, indicating more stable convergence of the improved framework. Qualitative visualizations demonstrate substantial reduction of missed detections in dense port scenarios, validating the practical value of the proposed edge-aware and shallow-guided fusion strategy for high-precision remote sensing small ship detection.

## Figures and Tables

**Table 1:** Ablation Study of EASA Module. Bold values indicate the best performance in each column.

Models	AP50	AP75	APs	APm	APl	mAP50–95
SHAB	0.841	0.636	0.229	**0.769**	0.900	0.594
GatedSHSA	0.845	**0.636**	0.240	0.767	0.908	0.594
EASA	**0.850**	0.635	**0.243**	0.767	**0.916**	**0.597**

**Table 2:** Performance Comparison of Different IoU Loss Functions. Bold values indicate the best performance in each column.

Models	AP50	AP75	APs	APm	APl	mAP50–95
GIoU (baseline)	0.833	0.629	0.227	0.765	0.911	0.585
CIoU	0.829	0.623	0.226	0.760	0.914	0.585
DIoU	**0.858**	0.637	0.228	**0.784**	0.920	0.597
EIoU	0.842	0.634	0.217	0.780	**0.920**	0.595
SIoU	0.855	0.635	0.232	0.770	0.884	0.593
MPDIoU	0.825	0.634	0.225	0.742	0.898	0.576
WIoUv3	0.847	**0.644**	**0.237**	0.771	0.915	**0.599**

**Table 3:** Independent evaluation of IoU loss variants on the Ships in Aerial Images test set. Bold values indicate the best performance in each column.

Models	AP50	AP75	APs	APm	APL	mAP50–95
GIoU (baseline)	0.204	0.047	**0.022**	0.152	0.245	0.087
CIoU	0.187	0.049	0.019	0.140	0.232	0.079
DIoU	0.200	0.059	0.021	0.163	0.249	0.085
EIoU	0.208	0.061	0.017	0.168	0.258	0.081
SIoU	0.212	0.060	0.019	0.144	0.293	0.089
MPDIoU	**0.215**	0.060	0.015	0.155	**0.296**	0.083
WIoUv3	0.206	**0.064**	**0.022**	**0.176**	0.278	**0.090**

**Table 4:** Experimental Environment Configuration.

Environment Configuration	Version
CPU	Intel(R) Xeon(R) Gold 6330
RAM	48G
GPU	RTX 3090(24GB)
CUDA	11.8
PyTorch	2.1.2
Python	3.10
Optimizer	AdamW
Learning rate	0.0004
Momentum	0.9
Weight decay	0.0001
Image size	640×640
Batch size	16
Epochs	132

**Table 5:** Comparison results among different models.

Models	AP50	AP75	APs	APm	APl	mAP50–95	FPS	Param (M)	GFLOPs
YOLO11	0.793	0.604	0.175	0.731	0.873	0.557	106.8	20.05	68.50
YOLO12	0.815	0.586	0.200	0.710	0.853	0.549	81.0	20.14	68.10
YOLO26	0.794	0.558	0.193	0.703	0.832	0.536	106.4	21.77	75.40
D-FINE-s	0.834	0.635	0.204	0.766	0.913	0.590	41.0	10.18	24.83
D-FINE-m	0.837	0.629	0.252	0.760	0.920	0.595	35.2	19.19	56.33
DEIM-s	0.846	0.637	0.231	0.779	0.926	0.602	39.2	10.18	24.82
RT-DETR-s	0.833	0.629	0.224	0.765	0.911	0.585	40.9	10.18	24.82
RT-DETR-m	0.820	0.636	0.201	0.764	0.908	0.584	34.6	19.19	56.33
RT-DETR-r18	0.785	0.607	0.209	0.723	0.861	0.543	53.9	19.88	59.86
FSDETR	0.827	0.640	0.235	0.756	0.918	0.562	65	14.7	-
CSFPR-RTDETR	0.840	0.643	0.239	0.770	0.905	0.581	35	14.9	63.9
Ours	0.858	0.651	0.256	0.782	0.931	0.613	37.6	12.96	43.42

**Table 6:** Ablation study of edge operators in EASA on the Ship-Detection dataset. Bold values indicate the best performance in each column.

Models	AP50	AP75	APs	APm	APl	mAP50–95
EASA (Scharr)	**0.850**	**0.635**	**0.243**	0.767	0.916	**0.597**
EASA (Sobel)	0.841	0.634	0.195	**0.779**	**0.926**	0.589
EASA (Learnable Scharr)	0.821	0.621	0.209	0.752	0.911	0.576

**Table 7:** Ablation Study of Proposed Components.

Models	AP50	AP75	APs	APm	APl	mAP50–95	FPS	Param (M)	GFLOPs
RT-DETR-s	0.833	0.629	0.224	0.765	0.911	0.585	40.9	10.18	24.82
+EASA	0.850	0.635	0.243	0.767	0.916	0.597	38.7	10.52	25.09
+SGDF	0.852	0.638	0.235	0.775	0.921	0.600	40.3	12.62	43.14
+WIoUv3	0.847	0.644	0.237	0.771	0.915	0.599	41.5	10.18	24.82
EASA + SGDF	0.852	0.648	0.246	0.777	0.925	0.606	37.1	12.96	43.42
SGDF + WIoUv3	0.852	0.655	0.249	0.774	0.920	0.605	39.6	12.62	43.14
EASA + WIoUv3	0.854	0.645	0.245	0.777	0.922	0.604	39.7	10.52	25.09
Ours	0.858	0.651	0.256	0.782	0.931	0.613	37.6	12.96	43.42

**Table 8:** Sensitivity analysis of λ in EASA on the Ship-Detection dataset. Bold values indicate the best performance in each column.

λ	AP50	AP75	APs	APm	APl	mAP50–95
0 (disabled)	0.823	0.625	0.220	0.747	0.883	0.573
0.01	0.834	0.625	0.226	0.766	0.906	0.590
0.02	0.836	0.629	0.231	0.768	**0.922**	0.593
0.03	0.848	0.630	0.233	0.769	0.913	0.587
0.05	0.827	0.613	0.208	**0.773**	0.911	0.581
0.10	0.826	0.630	0.205	0.772	0.918	0.584
Learnable (∼0.027)	**0.850**	**0.635**	**0.243**	0.767	0.916	**0.597**

Note: The row highlighted in yellow denotes the learnable parameter setting adopted in the proposed model.

**Table 9:** Sensitivity analysis of β in SGDF on the Ship-Detection dataset. Bold values indicate the best performance in each column.

β	AP50	AP75	APs	APm	APl	mAP50–95
0.1	0.841	0.637	0.214	0.773	0.916	0.591
0.2	0.846	0.633	0.228	**0.779**	0.913	0.596
0.3	0.848	**0.637**	0.232	**0.779**	0.914	0.595
0.4	0.840	0.633	0.213	0.775	**0.921**	0.594
0.5	0.845	0.631	0.218	0.772	0.913	0.588
Learnable (∼0.298)	**0.852**	**0.638**	**0.235**	0.775	**0.921**	**0.600**

Note: The row highlighted in yellow denotes the learnable parameter setting adopted in the proposed model.

**Table 10:** Generalization Results.

Models	AP50	AP75	APs	APm	APl	mAP50–95	Param (M)	GFLOPs
YOLO11	0.774	0.297	0.139	0.476	0.590	0.377	20.05	68.50
YOLO12	0.769	0.345	0.153	0.503	0.586	0.390	20.14	68.10
YOLO26	0.780	0.348	0.170	0.515	0.601	0.401	21.77	75.40
D-FINE-s	0.712	0.293	0.225	0.420	0.539	0.344	10.18	24.83
D-FINE-m	0.736	0.309	0.240	0.428	0.557	0.358	19.19	56.34
DEIM-s	0.722	0.281	0.240	0.437	0.507	0.349	10.18	24.82
RT-DETR-s	0.724	0.280	0.238	0.429	0.535	0.352	10.18	24.82
RT-DETR-m	0.747	0.314	0.247	0.443	0.554	0.363	19.19	56.33
RT-DETR-r18	0.610	0.225	0.203	0.342	0.413	0.280	19.88	59.86
FSDETR	0.742	0.303	0.239	0.435	0.531	0.355	14.7	-
CSFPR-RTDETR	0.715	0.313	0.232	0.411	0.504	0.344	14.9	63.9
Ours	0.751	0.320	0.245	0.445	0.559	0.367	12.96	43.42

**Table 11:** Cross-dataset generalization results on the Ships in Aerial Images test set. All models were trained on the primary dataset and evaluated without fine-tuning.

Models	AP50	AP75	APs	APm	APl	mAP50–95	Param (M)	GFLOPs
YOLO11	0.220	0.095	0.016	0.213	0.295	0.104	20.05	68.50
YOLO12	0.228	0.085	0.020	0.195	0.289	0.101	20.14	68.10
YOLO26	0.230	0.089	0.018	0.197	0.297	0.108	21.77	75.40
D-FINE-s	0.204	0.080	0.019	0.185	0.262	0.099	10.18	24.83
D-FINE-m	0.231	0.092	0.020	0.200	0.348	0.112	19.19	56.34
DEIM-s	0.221	0.098	0.019	0.223	0.288	0.112	10.18	24.82
RT-DETR-s	0.220	0.082	0.021	0.196	0.263	0.104	10.18	24.82
RT-DETR-m	0.231	0.088	0.023	0.197	0.327	0.111	19.19	56.33
RT-DETR-r18	0.216	0.083	0.019	0.201	0.272	0.100	19.88	59.86
FSDETR	0.228	0.093	0.023	0.208	0.314	0.108	14.7	-
CSFPR-RTDETR	0.230	0.089	0.022	0.208	0.316	0.113	14.9	63.9
Ours	0.241	0.096	0.025	0.212	0.332	0.117	12.96	43.42

**Table 12:** Baseline RT-DETR-s Stability across Random Seeds.

Run	AP50 (%)	AP75 (%)	APs (%)	mAP50–95 (%)
1	84.5	63.1	21.8	58.8
2	83.0	61.2	22.9	58.4
3	83.3	62.9	22.4	58.5
4	83.8	65.0	23.0	59.2
Mean	83.65	63.05	22.53	58.73
Std	0.568	1.346	0.487	0.311
Min–Max	mAP50–95: 58.4–59.2			
Range	mAP50–95: 0.8			

**Table 13:** Proposed Model Stability across Random Seeds.

Run	AP50 (%)	AP75 (%)	APs (%)	mAP50–95 (%)
1	85.5	64.8	25.2	61.0
2	85.3	64.8	24.9	60.7
3	85.8	65.1	25.6	61.3
4	85.4	64.6	25.5	60.9
Mean	85.5	64.8	25.3	60.98
Std	0.187	0.178	0.274	0.217
Min–Max	mAP50–95: 60.7–61.3			
Range	mAP50–95: 0.6			

## Data Availability

The Ship Detection from Aerial Images dataset used for the main experiments (comparison, ablation and stability tests) and the remote-ship dataset used for generalization evaluation are both publicly available optical remote sensing ship detection benchmarks. The Ship Detection from Aerial Images dataset can be downloaded from https://www.kaggle.com/datasets/andrewmvd/ship-detection (accessed on 20 July 2026), the remote-ship dataset is accessible at https://www.kaggle.com/datasets/hhy1998/remote-ship (accessed on 16 July 2026), and the Ships in Aerial Images dataset is accessible at https://www.kaggle.com/datasets/siddharthkumarsah/ships-in-aerial-images (accessed on 8 September 2026).
